# Overview of the organization of protease genes in the genome of *Leishmania* spp

**DOI:** 10.1186/1756-3305-7-387

**Published:** 2014-08-20

**Authors:** Mariana Silva-Almeida, Franklin Souza-Silva, Bernardo Acácio Santini Pereira, Michelle Lopes Ribeiro-Guimarães, Carlos Roberto Alves

**Affiliations:** Laboratório de Biologia Molecular e Doenças Endêmicas, Instituto Oswaldo Cruz, Fundação Oswaldo Cruz, Av. Brasil 4365, Rio de, Janeiro, CEP 21040-900 Brasil

**Keywords:** *Leishmania*, *Leishmania (Viannia) braziliensis*, *Leishmania (Leishmania) infantum*, *Leishmania (Leishmania) major*, *Leishmania (Leishmania) mexicana*, Proteases

## Abstract

**Background:**

The genus *Leishmania* includes protozoan parasites that are able to infect an array of phlebotomine and vertebrate species. Proteases are related to the capacity of these parasites to infect and survive in their hosts and are therefore classified as virulence factors.

**Findings:**

By analyzing protease genes annotated in the genomes of four *Leishmania* spp [*Leishmania (Leishmania) infantum, L. (L.) major, L. (L.) mexicana* and *L. (Viannia) braziliensis*], these genes were found on every chromosome of these protozoa. Four protease classes were studied: metallo-, serine, cysteine and aspartic proteases. Metalloprotease genes predominate in the *L. (V.) braziliensis* genome, while in the other three species studied, cysteine protease genes prevail. Notably, cysteine and serine protease genes were found to be very abundant, as they were found on all chromosomes of the four studied species. In contrast, only three aspartic protease genes could be detected in these four species. Regarding gene conservation, a higher number of conserved alleles was observed for cysteine proteases (42 alleles), followed by metalloproteases (35 alleles) and serine proteases (15 alleles).

**Conclusions:**

The present study highlights substantial differences in the organization of protease genes among *L. (L.) infantum, L. (L.) major, L. (L.) mexicana* and *L. (V.) braziliensis*. We observed significant distinctions in many protease features, such as occurrence, quantity and conservation. These data indicate a great diversity of protease genes among *Leishmania* species, an aspect that may be related to their adaptations to the peculiarities of each microenvironment they inhabit, such as the gut of phlebotomines and the immune cells of vertebrate hosts.

**Electronic supplementary material:**

The online version of this article (doi:10.1186/1756-3305-7-387) contains supplementary material, which is available to authorized users.

## Background

The World Health Organization classifies the leishmaniases, infections caused by parasites of the genus *Leishmania*, among emerging diseases that lack effective control. Annually, an estimated 1.3 million new cases occur and 20,000 to 30,000 deaths are attributed to these diseases [1]. The clinical forms range in severity and are classified as follows: punctuate skin lesions to oronasal disfigurement are classified as cutaneous leishmaniasis (CL), whereas fatal systemic infections are classified as visceral leishmaniasis (VL). *Leishmania* spp are distributed worldwide and are organized into subgenera and species complexes. Their transmission to mammalian hosts occurs during the blood meal of infected sandflies, which in turn acquire the parasites when feeding on an infected host, thus maintaining the cycle of the disease. The species grouped into the *Leishmania* (*Leishmania*) *donovani* complex, including *L.* (*L*.) *infantum*, are the agents of VL. As for the species commonly associated with CL, *L.* (*L*.) *major* is reported in the Old World, whereas *L.* (*L*.) *mexicana* and *L.* (*Viannia*) *braziliensis* are the main species reported in the New World. This latter species is also associated with the mucocutaneous form of the disease.

In a recent review study, we have highlighted the pivotal roles of proteases as virulence factors for *Leishmania* spp [2]. Such enzymes have been implicated in many parasitic activities, such as tissue invasion, survival in macrophages and host immune response modulation.

Proteases are classified according to their physicochemical features as: optimal pH for activity, kind of catalytic activity, nature of catalytic site and homology with reference structure [3]. According to the enzymatic nomenclature committee, the Joint Commission on Biochemical Nomenclature (JCBN), peptidases are allocated into the Enzyme Class (EC) 3 (hydrolases) and subclass 3.4 (peptidases). They can be subdivided into exopeptidases (EC 4.11 - EC.4.19) and endopeptidases (EC 3.4.21 - 3.4.25), and the latter are organized according to the amino acids related to catalysis and the nature of catalytic site [4]. In addition, endopeptidases are further divided into classes according to the main catalytic mechanism involved in their hydrolytic activities, e.g., serine, threonine, aspartate, metallo- and cysteine proteases [4].

The aim of the present study is to analyse the genomic organization of proteases in four *Leishmania* species known to cause disease in humans: *L*. (*V*.) *braziliensis*, *L*. (*L*.) *major*, *L*. (*L*.) *mexicana* and *L*. (*L*.) *infantum*, and, concomitantly, to evaluate their diversity among these species. Due to the importance of these enzymes in the life cycle of these parasites, the genomic data gathered here would be very useful as a basis for further studies correlating infection characteristics of each of the studied species with their protease richness. Understanding how these enzymes are organized and conserved (or diverged) in the different *Leishmania* subgenera and species is very useful in helping to identify new targets with the most potential for chemotherapy or vaccination strategies.

## Findings and discussion

We performed a comparative genomic analysis on the organization of protease genes in four species, a methodology we applied to identify species-specific features that may account for phenotypic or virulence differences among the studied species. Gene divergence, acquisition, loss, and rearrangement within and between syntenic regions have shaped the genomes of the trypanosomatids [5] and can explain the organization and diversity of the degradome (the complete set of protease genes encoded by the genome of a certain organism) of *Leishmania* spp [6]. Initially, we performed a survey of the predicted protease sequences present in the annotated genomes of *L. (V.) braziliensis*, *L. (L.) major, L. (L.) mexicana* and *L. (L.) infantum* in the GeneDB genome database [7]. This survey was conducted using the following keywords: protease, peptidase, proteinase, aspartic protease, cysteine protease, serine protease and metalloprotease.

In an initial analysis of the data retrieved by the methodology above, the abundance of protease genes in the genomes of each of the studied species was defined. While protease genes account for 2.18% of the total genes in *L. (V.) braziliensis*, these genes account for smaller percentages in the other species: 1.61% in *L. (L.) infantum*, 1.52% in *L. (L.) mexicana* and 1.41% in *L. (L.) major*.

**Figure 1:**
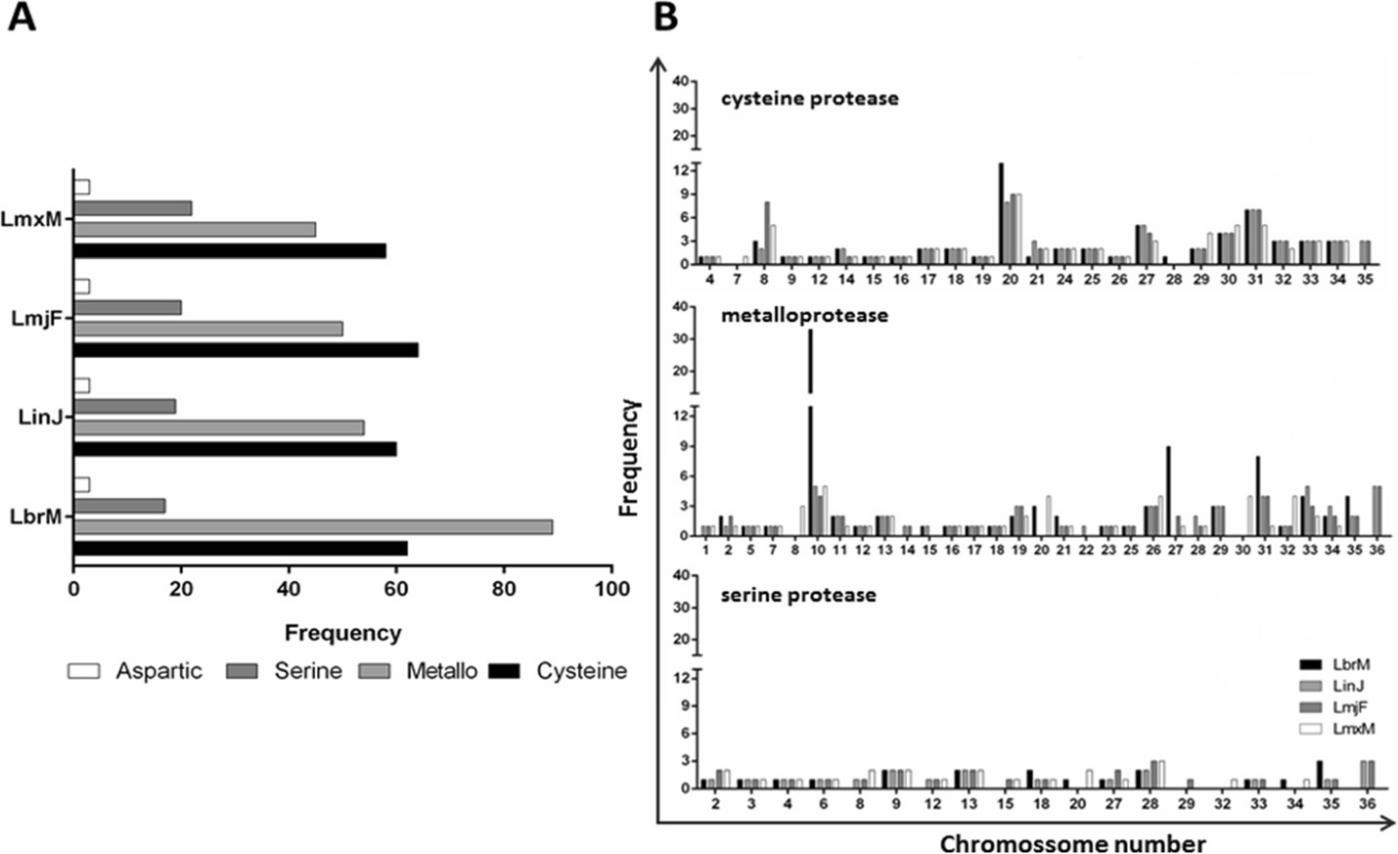
**Annotated gene sequences of proteases in the genome of** ***Leishmania spp.*** **(A)** Number of protease gene in *Leishmania* spp. **(B)** Frequency of chromosome in different classes of proteases in *Leishmania* spp. LbrM - *L. (V.) braziliensis*, LinJ - *L. (L.) infantum*, LmjF - *L. (L.) major,* LmxM - *L. (L.) mexicana.*

Metalloprotease genes predominate in *L. (V.) braziliensis*, while in the other species the cysteine protease genes prevail. Our analysis showed that 52% of the protease genes in *L. (V.) braziliensis* are metalloproteases and this same class accounts for 40% of protease genes in *L. (L.) infantum* and 35% in *L. (L.) major* and *L. (L.) mexicana*. The percentages of cysteine and serine protease gene are close among the studied species: cysteine protease genes represent 36 to 47% of the total protease genes, whereas serine protease genes represent 10 to 16%. Very few aspartic protease genes were identified, amounting to only three in each of the four species (Figure 1).

A very interesting finding we observed is that protease genes are present in every chromosome of the studied *Leishmania* spp, but occur in different frequencies (Figure 1). This discovery is consistent with the previously reported importance of proteases for these parasitic organisms, as it reveals that genes encoding these enzymes are abundantly scattered among the *Leishmania* spp genomes, and is, complementarily, an indication of the distinct pattern evolution has impinged over the different species.

Other studies regarding gene organization in *Leishmania* spp have been conducted before and related the structural configuration of the genes with important functional features. The organization of genes in tandem repeats allows parasites to quickly generate a high number of transcripts that may be needed in large amounts [8]. Other authors hypothesise that *Leishmania* spp. might have a strategy to increase mRNA levels by duplicating genes on disomic chromosomes or by forming supernumerary chromosomes [9].

Of the chromosomes that we identified as containing metalloprotease genes, 18 are common for all studied species. Notably, the presence of metalloprotease genes on chromosomes 8 and 30 is exclusive to *L. (L.) mexicana*. Similar exclusiveness for the presence of metalloprotease genes was observed for chromosome 22 in *L. (L.) infantum* and chromosome 20 in *L. (V.) braziliensis*. Cysteine protease genes are present in 22 chromosomes common to all four species studied. Cysteine protease genes are also present on chromosome 7 exclusively in *L. (L.) mexicana*, on chromosome 28 in *L. (V.) braziliensis* and on chromosome 35 in *L. (L.) infantum* and *L. (L.) major*. Serine protease genes are present in 9 chromosomes common to all four species studied and the number of these genes does not exceed three per chromosome. The presence of genes for this protease class on chromosome 29 is exclusive to *L. (L.) major* and on chromosome 20 to *L. (V.) braziliensis.* The protease class found to have the fewest coding genes was aspartic proteases: only three genes for this class were observed, but the chromosomes on which they are present are common to all studied species. These genes are located on chromosomes 1, 15 and 29 (Figure 1).

Regarding genes for different protease classes that occur on the same chromosome, most of the studied chromosomes were found to contain genes for multiple protease classes. The exceptions were chromosomes 3 and 6, which were found to contain only serine protease genes and chromosomes 5, 11 and 22, which were found to contain only metalloprotease genes.

Due to fusion events that occurred in *Leishmania* chromosomes, we observed an interesting pattern of organization of protease genes where the same arrangement of alleles is maintained across different species but is located on different chromosomes. Graphical representations of such fusion events were developed using the Artemis and ACT software [10] (Additional file 1: Figure S1 to S8).

**Figure 2:**
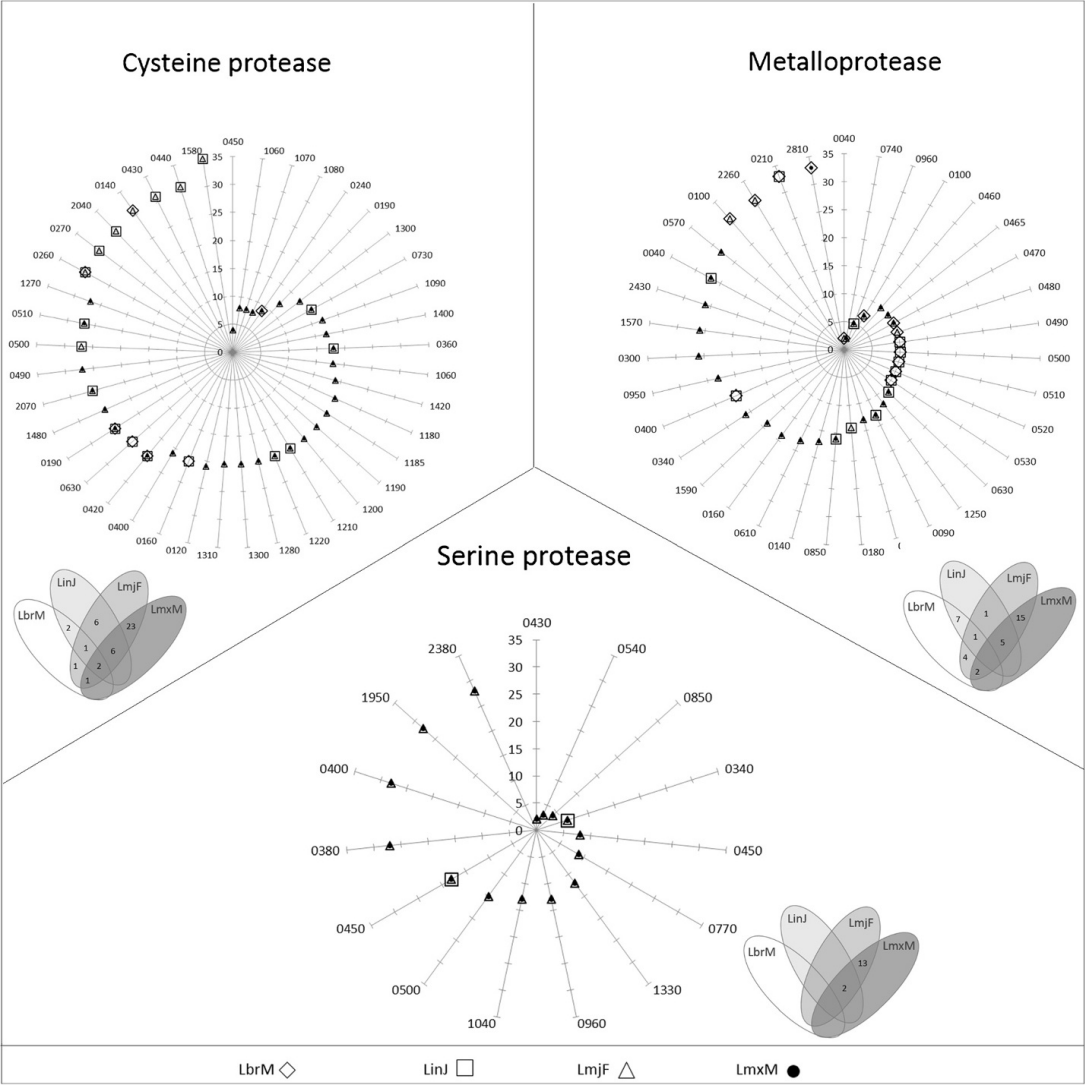
**Analysis of conserved alleles in the genome of** ***Leishmania spp.*** Radar charts indicate the conserved alleles on the periphery and the chromosome number on the y axis, while in the Venn diagrams the number of conserved alleles in *Leishmania* spp is informed. LbrM - L. (V.) braziliensis, LinJ - *L. (L.) infantum*, LmjF - L. (L.) major, LmxM - *L. (L.) mexicana*.

Nevertheless, there is a trend of conservation of some alleles in the same chromosomes across the studied *Leishmania* species. We observed 42 conserved alleles of cysteine proteases, 35 of metalloproteases and 15 of serine proteases (Figure 2). The conserved alleles are predominantly grouped on chromosome 10 for cysteine proteases, chromosome 30 for metalloproteases and chromosome 28 for serine proteases.

Among all the analysed protease genes, only two alleles were found to be conserved on the same chromosome for all four species: alleles of cysteine protease genes coding for ubiquitin carboxyl-terminal hydrolases (Clan CA, family C12) located on chromosomes 24 and 25 (alleles 0420 and 0190, respectively) of all species.

Notably, *L.* (*L.*) *major* and *L.* (*L.*) *mexicana* were found to show more synteny than the other species, containing 23, 15 and 13 conserved alleles for cysteine, metallo- and serine proteases, respectively. Conversely, *L. (V.) braziliensis* was not found to show synteny for serine protease genes of any other species. Although this absence of synteny was observed in the only species in our analysis classified into a different subgenus, it has been proposed by Peacock *et al.* [11] that such absence would not necessarily indicate a lineage-specific diversity in *Leishmania* spp.

One of the first comparative genomic studies of *Leishmania* showed that despite phenotypic variations among species, only a few genes are truly species-specific [11]. In agreement with such reports, we also observed few genes that do not show similarity to any others. They show sequence identities lower than 80% to other genes (Additional file 2: Table S1). This is an important finding, as these exclusive genes can help explain why these species cause different forms of diseases and are present in specific vectors and hosts. Previously, it was reported that more than 99% of genes are conserved between *L. (V.) braziliensis*, *L. (L.) infantum* and *L. (L.) major*, revealing a high degree of synteny for genomes of different *Leishmania* species [11]. Our data indicates that, when analysing strictly protease genes, this same scenario holds up, as we also observed high synteny between the studied species.

When contemplating the usefulness of parasite proteases as new targets for chemotherapies, it is very important to consider the hypothesis that these enzymes are unique to the *Leishmania* species and quite different from corresponding enzymes in their mammalian hosts, such as humans and dogs. Thus, to verify this hypothesis, we conducted a BLAST (Basic Local Alignment Search Tool) analysis to compare the genes that show synteny among the greater number of the four species (represented in the intersection of the Venn diagram – Figure 2) with mammalian protease genes (taxid:40674). The genes 05.0960 and 11.0630 of *L. (L.) major*, *L. (L.) mexicana*, *L. (L.) major* show the highest degree of relational similarity with mammalian genes, with approximately 69% sequence identity and a query coverage of up to 39%. However, in general, the query coverage was very low, with a mean value of 2%. In addition, to perform a similar study with other proteases that did not show synteny among all the studied species, we used a different approach.

Initially, a multiple alignment analysis was carried out on the sequences of protease genes of the four species (software *CD-HIT* [12]), using a cutoff of 80% sequence identity to cluster them. As result, we were able to establish 28 clusters of metalloprotease genes, 27 of cysteine protease genes, 11 of serine protease genes and 1 of aspartic protease genes.

The consensus sequences (Additional file 2: Table S2) of each cluster were then used in the BLAST analysis to find similarity with mammalian genes. We identified sequences of O-sialoglycoprotein endopeptidase genes of hamster, dog, wolf and mice with 69% sequence identity to a consensus sequence of *Leishmania* metalloprotease genes LbrM.31.0100, LinJ.31.0110, LmjF.31.0100 and LmxM.31.0100. Sequences of 26S subunit ATPase genes of a lagomorph *Ochotona princeps* and of mice showed 65% sequence identity to a consensus sequence of serine protease genes LbrM.03.0450, LinJ.03.0520, LmjF.03.0540 and LmxM.03.0540. Additionally, we could not find any similarity among sequences of cysteine and aspartic protease genes of mammals and *Leishmania* spp.

**Figure 3:**
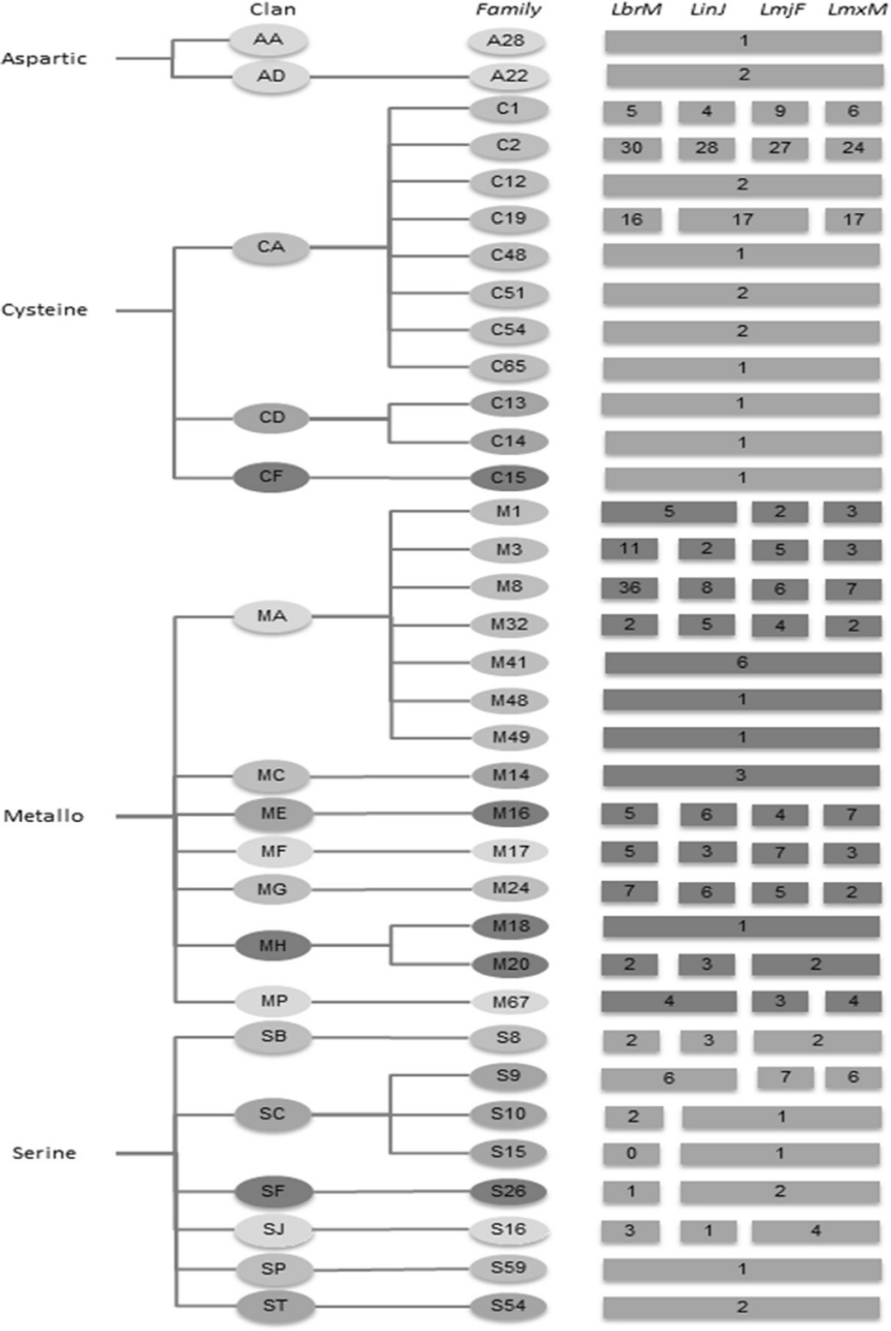
**Clans and families of** ***Leishmania*** **spp. proteases.** Classification of proteases sequence researched. Nomenclature is based on the MEROPS database (December 20013, release 9.10 - http://merops.sanger.ac.uk/). Numbers in squares represent the estimated number of protease gene in each family for each species, as number in rectangle corresponds to the same number of protease per specie. LbrM - *L. (V.) braziliensis*, LinJ - *L. (L.) infantum*, LmjF - *L. (L.) major,* LmxM - *L. (L.) mexicana*.

As proteases can be grouped into different families and clans depending on intrinsic evolutionary relationships, we classified and organized the protease genes surveyed in this study applying criteria from MEROPS [13] (up to December 2013) (Figure 3). This classification is based on structural and functional similarities between these proteolytic enzymes. Clans contain enzymes with related structures and families contain enzymes with related sequences [4]. This classification is highly relevant to understanding the organization of these parasites’ degradomes.

Cysteine proteases and metalloproteases are the major representative classes of proteases in this study, corresponding to 43% and 42%, respectively of the protease genes in the studied *Leishmania* spp. In this survey, three clans of cysteine proteases were observed in the studied species: clan CA, CD and CF. These cysteine proteases are distributed among 11 families from which C1, C2 and C19 have more members. The MPs observed in the study belong to the clans MA, MC, ME, MF, MG, MH and MP and are further distributed among 14 families (Figure 3). The diversity of protease genes observed in the analysis reinforces the idea that this class of enzyme is crucial to the parasite lifecycle, although until now the role of most of these proteases can only be predicted based on current knowledge of homologous enzymes, therefore pointing to the necessity of more studies characterising proteases [14].

The high number of metalloprotease genes in *L. (V.) braziliensis* relates to the 36 distinct genes of the zinc metalloprotease gp63. This metalloprotease is a very well-characterised virulence factor for *L. (L.) braziliensis* and has several reported functions in the interactions of this parasite with its hosts [15]. In *L. (L.) major, L. (L.) mexicana* and *L. (L.) infantum*, the diversity of gp63 genes is much lower: only 6, 7 and 8 genes, respectively, of this protease could be found (Figure 3). The organization of metalloprotease genes in species of the subgenus *Viannia* is rather different than that of species of the subgenus *Leishmania* [16]. The predominance of metalloprotease genes in *L. (V.) braziliensis,* a peculiarity also observed in *L. (V.) guyanensis* [17], has a biological significance not completely understood [8, 18]. Amplification of genes is a common phenomenon in *Leishmania* [19–21] and is a likely source of the differences between the two subgenera. Such interesting variation might have fundamental implications for the way each species interacts with its hosts.

Our study highlights the informative potential of analysing genome databases for understanding the gene organization of parasites. However, one should be aware that not all annotated proteases have described roles in the *Leishmania* life cycle. Thus, the picture observed here is not yet complete.

It is still unclear how the current organization of *Leishmania* spp genomes evolved, but the set of results gathered here emphasises the capacity of *Leishmania* species to use the plasticity of their genomes to modulate their phenotypes and increase their odds of survival within hosts, among other biological processes. The diversity of protease genes described by our present study points to their potential importance as survival and adaptation tools and, consequently, as important targets in vaccination and therapy strategies.

## Electronic supplementary material

Additional file 1: Figure S1: Representation of fusion events between chromosomes 29 and 8 of *L. (L.) major* (LmjF) and *L. (L.) mexicana* (LmxM), respectively. **Figure S2.** Representation of allelic transpositions between chromosomes 30 and 29 of *L. (L.) major* (LmjF) and *L. (L.) mexicana* (LmxM), respectively. **Figure S3.** Representation of allelic transpositions between chromosomes 31 and 30 of *L. (L.) major* (LmjF) and *L. (L.) mexicana* (LmxM), respectively. **Figure S4.** Representation of allelic transpositions between chromosomes 32 and 31 of *L. (L.) major* (LmjF) and *L. (L.) mexicana* (LmxM), respectively. **Figure S5.** Representation of allelic transpositions between chromosomes 33 and 32 of *L. (L.) major* (LmjF) and *L. (L.) mexicana* (LmxM), respectively. **Figure S6.** Representation of allelic transpositions between chromosomes 34 and 33 of *L. (L.) major* (LmjF) and *L. (L.) mexicana* (LmxM), respectively. **Figure S7.** Representation of allelic transpositions between chromosomes 35 and 34 of *L. (L.) major* (LmjF) and *L. (L.) mexicana* (LmxM), respectively. **Figure S8.** Representation of fusion events between chromosomes 36 and 20 of *L. (L.) major* (LmjF) and *L. (L.) mexicana* (LmxM), respectively. (PDF 732 KB)

Additional file 2: Table S1: Protease genes exclusive to each *Leishmania* sp. amongst the four studied species. **Table S2.** Cluster *of genes.* (DOCX 43 KB)
