# Supplementary material for: Overview of the organization of protease genes in the genome of Leishmania spp
Source: Parasit Vectors. 2014 Aug 20;7:387. doi: 10.1186/1756-3305-7-387 (PMC4158035; doi:10.1186/1756-3305-7-387)
Supplement: Supplementary file 1 — Additional file 1: Figure S1: Representation of fusion events between chromosomes 29 and 8 of L. (L.) major (LmjF) and L. (L.) mexicana (LmxM), respectively. Figure S2. Representation of allelic transpositions between chromosomes 30 and 29 of L. (L.) major (LmjF) and L. (L.) mexicana (LmxM), respectively. Figure S3. Representation of allelic transpositions between chromosomes 31 and 30 of L. (L.) major (LmjF) and L. (L.) mexicana (LmxM), respectively. Figure S4. Representation of allelic transpositions between chromosomes 32 and 31 of L. (L.) major (LmjF) and L. (L.) mexicana (LmxM), respectively. Figure S5. Representation of allelic transpositions between chromosomes 33 and 32 of L. (L.) major (LmjF) and L. (L.) mexicana (LmxM), respectively. Figure S6. Representation of allelic transpositions between chromosomes 34 and 33 of L. (L.) major (LmjF) and L. (L.) mexicana (LmxM), respectively. Figure S7. Representation of allelic transpositions between chromosomes 35 and 34 of L. (L.) major (LmjF) and L. (L.) mexicana (LmxM), respectively. Figure S8. Representation of fusion events between chromosomes 36 and 20 of L. (L.) major (LmjF) and L. (L.) mexicana (LmxM), respectively. (PDF 732 KB) [file 13071_2014_1574_MOESM1_ESM.pdf]

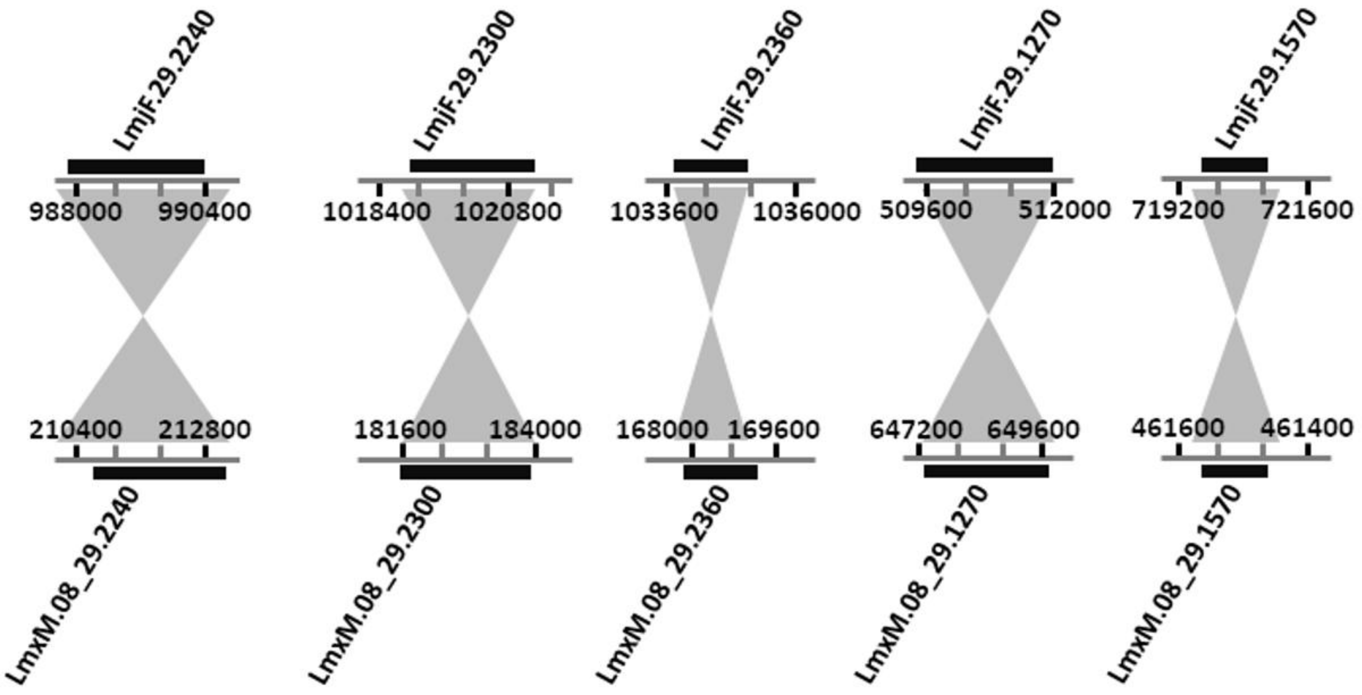

Supplementary figure 1: Representation of fusion events between chromosomes 29 and 8 of *L. (L.) major* (LmjF**)** and *L. (L.) mexicana* (LmxM), respectively.

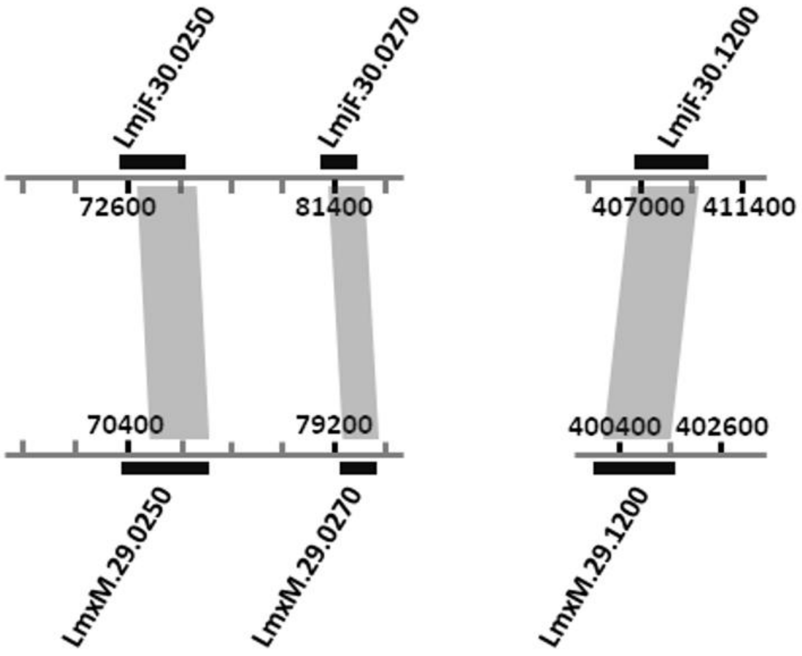

Supplementary figure 2: Representation of allelic transpositions between chromosomes 30 and 29 of *L. (L.) major* (LmjF**)** and *L. (L.) mexicana* (LmxM), respectively.

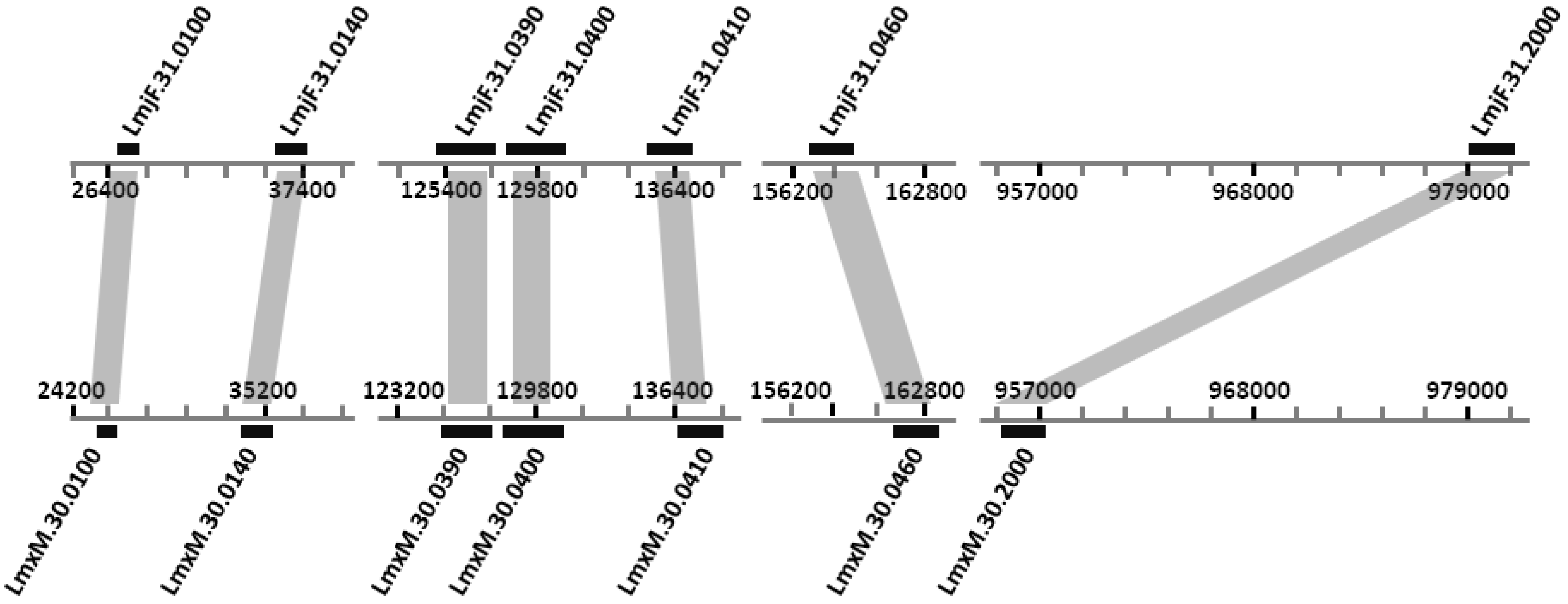

Supplementary figure 3: Representation of allelic transpositions between chromosomes 31 and 30 of *L. (L.) major* (LmjF**)** and *L. (L.) mexicana* (LmxM), respectively.

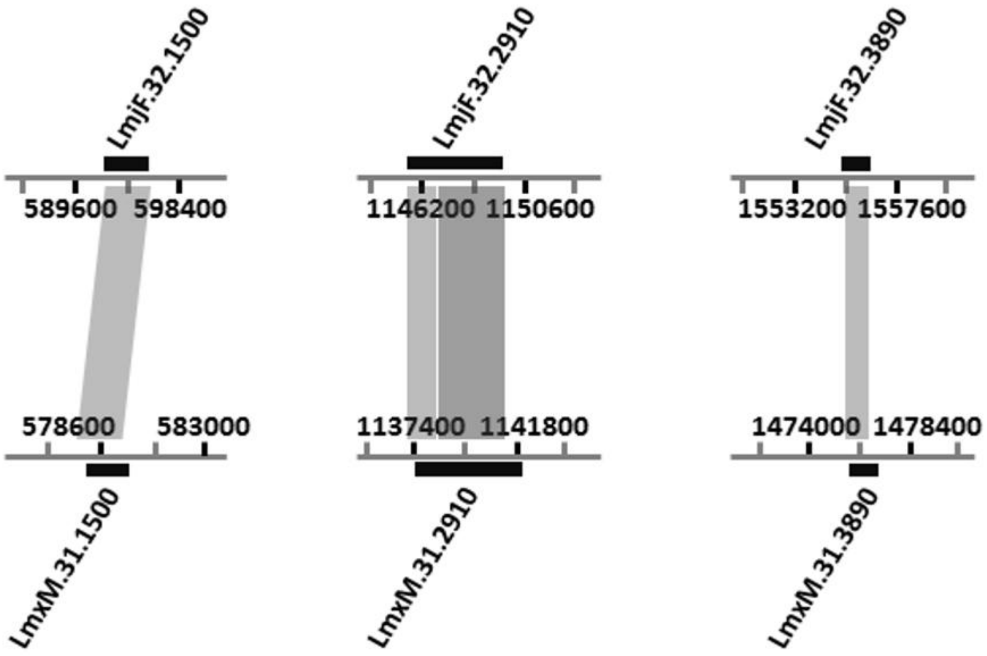

Supplementary figure 4: Representation of allelic transpositions between chromosomes 32 and 31 of *L. (L.) major* (LmjF**)** and *L. (L.) mexicana* (LmxM), respectively.

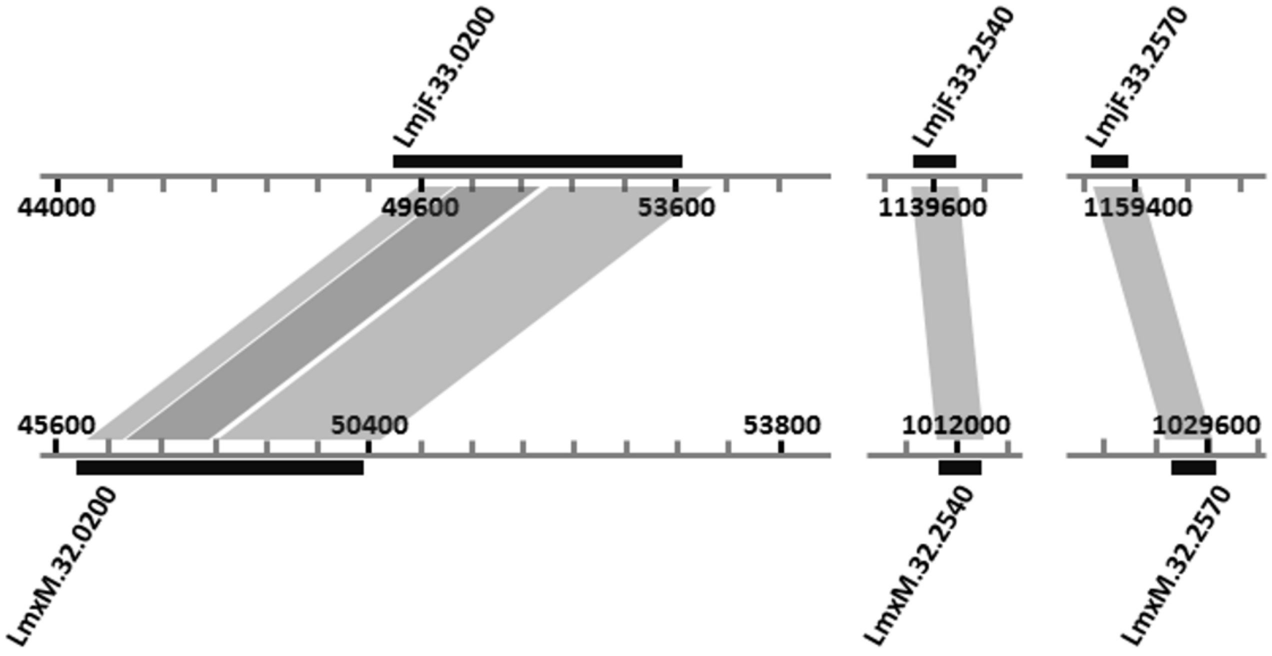

Supplementary figure 5: Representation of allelic transpositions between chromosomes 33 and 32 of *L. (L.) major* (LmjF**)** and *L. (L.) mexicana* (LmxM), respectively.

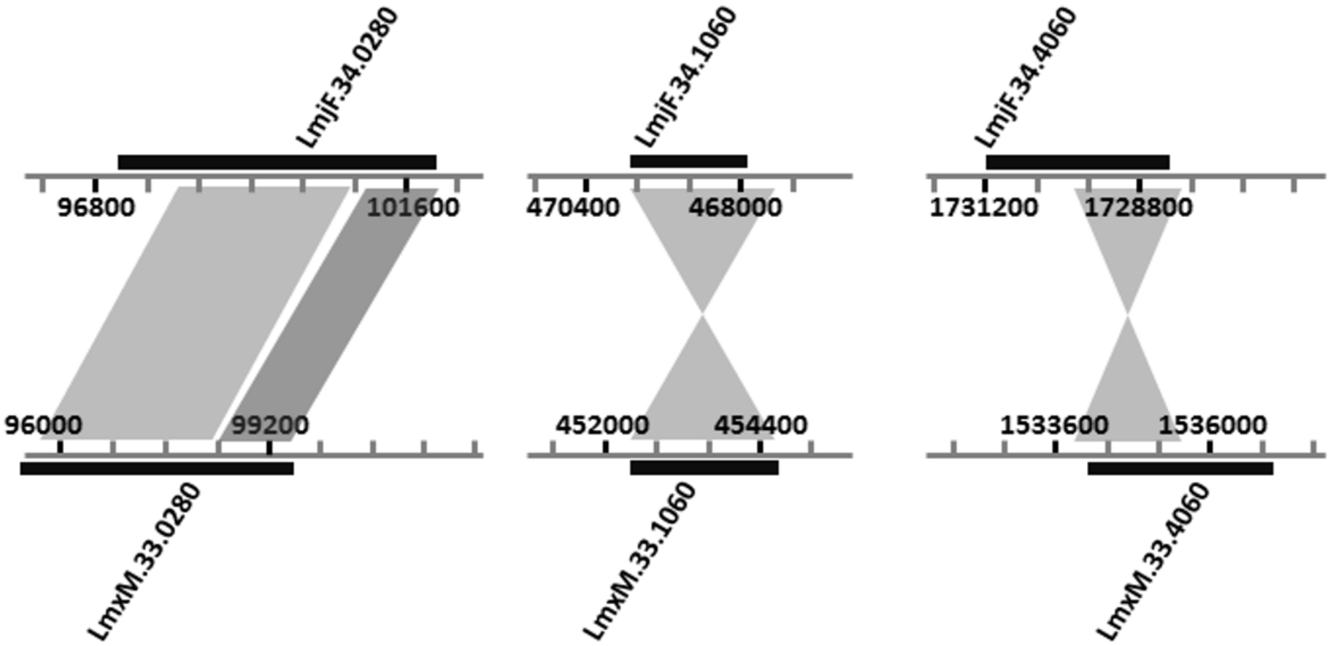

Supplementary figure 6: Representation of allelic transpositions between chromosomes 34 and 33 of *L. (L.) major* (LmjF**)** and *L. (L.) mexicana* (LmxM), respectively.

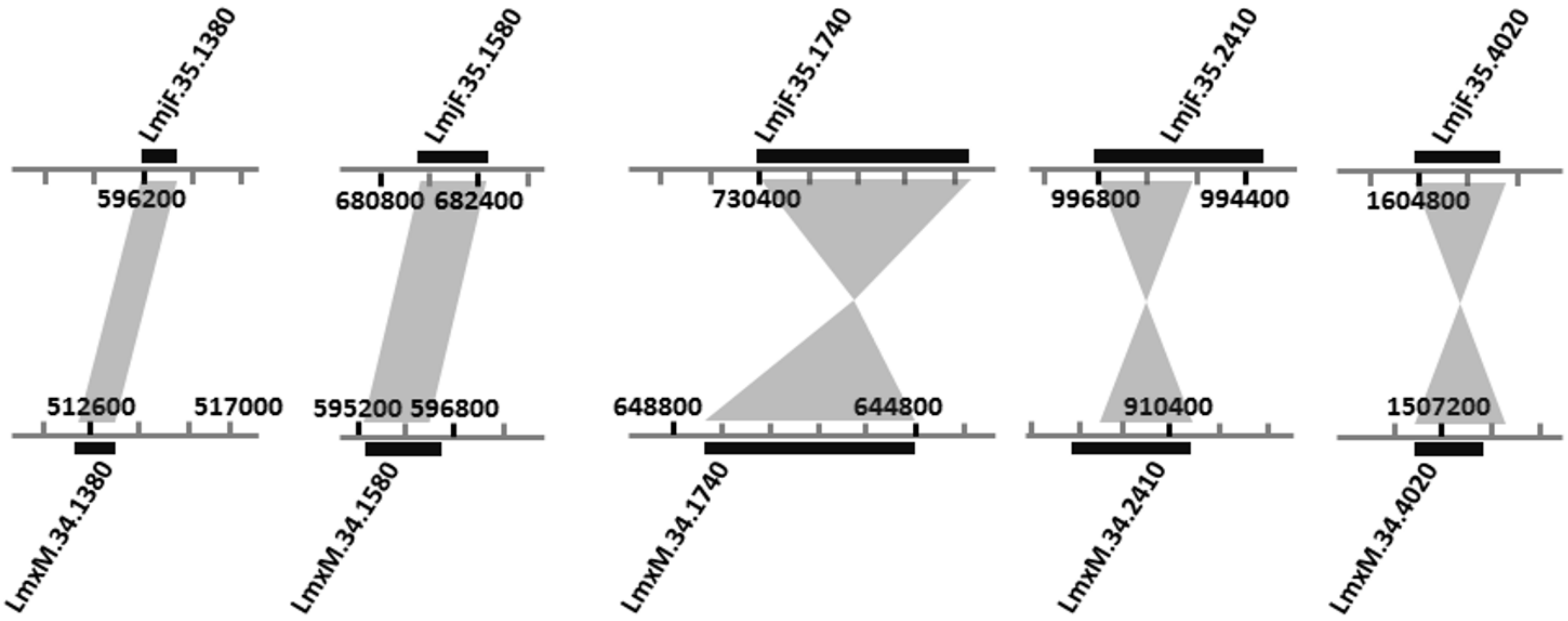

Supplementary figure 7: Representation of allelic transpositions between chromosomes 35 and 34 of *L. (L.) major* (LmjF**)** and *L. (L.) mexicana* (LmxM), respectively.

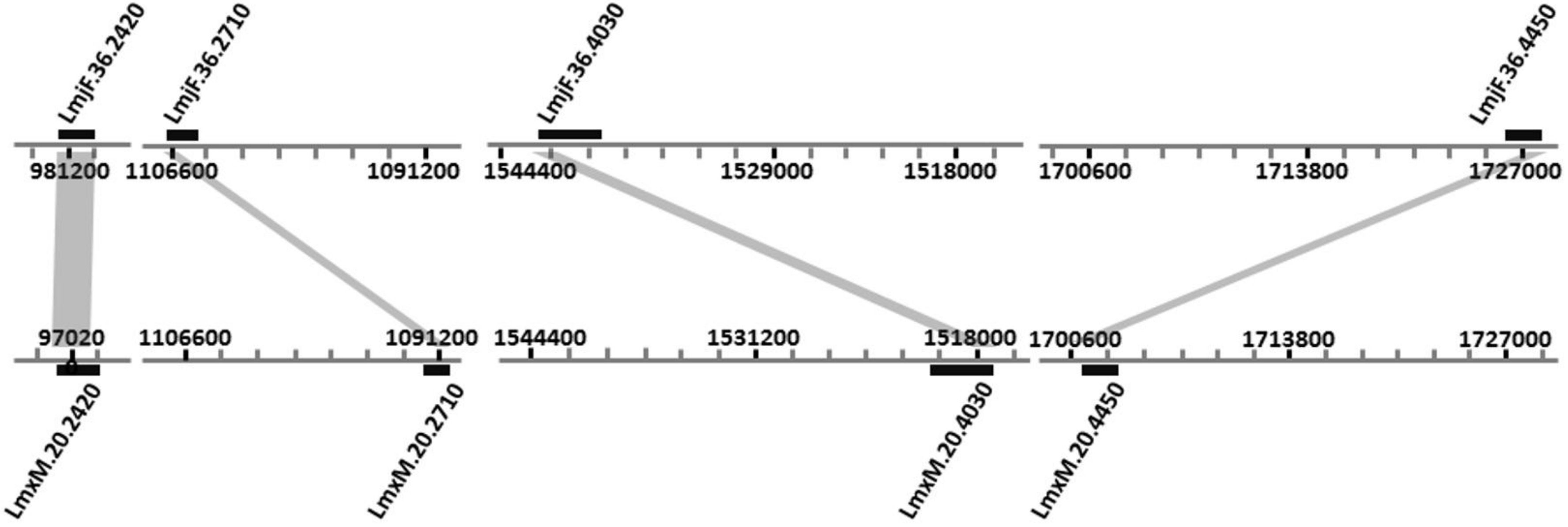

Supplementary figure 8: Representation of fusion events between chromosomes 36 and 20 of *L. (L.) major* (LmjF**)** and *L. (L.) mexicana* (LmxM), respectively.
