# Supplementary material for: Overview of the organization of protease genes in the genome of Leishmania spp
Source: Parasit Vectors. 2014 Aug 20;7:387. doi: 10.1186/1756-3305-7-387 (PMC4158035; doi:10.1186/1756-3305-7-387)
Supplement: Supplementary file 2 — Additional file 2: Table S1: Protease genes exclusive to each Leishmania sp. amongst the four studied species. Table S2. Cluster of genes. (DOCX 43 KB) [file 13071_2014_1574_MOESM2_ESM.docx]

| **Suplemmentary table 1:** Protease genes exclusive to each *Leishmania* sp. amongst the four studied species. | **Protease** | ***Leishmania spp*** | | |  |
| --- | --- | --- | --- | --- | --- |
|  |  | ***Leishmania (V.) braziliensis*** | ***Leishmania (L.) mexicana*** | ***Leishmania (L.) infantum*** |  |
| **cysteine protease** | calpain-like cysteine peptidase | LbrM.28.2100; LbrM.20.5340; LbrM.20.5430; LbrM.27.0600; LbrM.27.2140; LbrM.30.1980; LbrM.31.0590 | LmxM.20.1185 LmxM.20.1300 LmxM.27.0510 LmxM.29.2040 LmxM.31.0970 LmxM.31.2010 |  |  |
|  |  |  |  |  |  |
|  |  |  |  |  |  |
|  |  |  |  |  |  |
|  |  |  |  |  |  |
|  |  |  |  |  |  |
|  | ubiquitin hydrolase | LbrM.09.0240; LbrM.12.0210; LbrM.15.1240; LbrM.16.0720; LbrM.17.1200; LbrM.24.0630; LbrM.34.2360 |  | LinJ.32.1310 | |
|  |  |  |  |  |  |
|  |  |  |  |  |  |
|  |  |  |  |  |  |
|  | D-alanyl-glycyl endopeptidase-like protein | LbrM.33.3140; LbrM.33.3110 |  |  |  |
|  |  |  |  |  |  |
|  | cysteine peptidase A | LbrM.19.1690 |  |  |  |
|  | cysteine peptidase C | LbrM.29.0850 |  |  |  |
|  | cytoskeleton-associated protein CAP5.5 | LbrM.31.0600 |  |  |  |
|  | SUMO1/Ulp2 | LbrM.26.1990 |  |  |  |
|  | cysteine peptidase B |  | LmxM.07.0550 |  |  |
| **metalloprotease** | methionine aminopeptidase | LbrM.21.0950 |  |  |  |
|  | zinc carboxypeptidase | LbrM.35.4270 |  |  |  |
|  | aminopeptidase P1 | LbrM.35.5830 | LmxM.36.5560 |  |  |
|  | Aminopeptidase | LbrM.31.2530; LbrM.31.2610 |  |  |  |
|  | peptidase M20/M25/M40 |  |  | LinJ.33.1710 |  |
|  | peptidyl dipeptidase | LbrM.02.0670; LbrM.27.1640  LbrM.27.1650; LbrM.27.2770  LbrM.27.2780; LbrM.27.2800  LbrM.27.2820; LbrM.27.2840 |  |  |  |
|  | Leishmanolysin (GP63-1) | LbrM.10.0470; LbrM.10.0480  LbrM.10.0500; LbrM.10.0520  LbrM.10.0540; LbrM.10.0570  LbrM.10.1570; LbrM.10.1590  LbrM.10.1610; LbrM.10.1620  LbrM.10.1630; LbrM.10.1640  LbrM.10.1650; LbrM.10.1660  LbrM.10.1670; LbrM.10.1680  LbrM.10.1710; LbrM.10.1720 |  |  |  |
|  | Leishmanolysin (GP63-2) | LbrM.10.1550; LbrM.10.1560  LbrM.10.0490; LbrM.10.1700 |  |  |  |
|  | Leishmanolysin (GP63-3) | LbrM.10.0510; LbrM.10.0530  LbrM.10.0550; LbrM.10.0560  LbrM.10.0580; LbrM.10.0590  LbrM.10.0600; LbrM.10.0610  LbrM.10.1540; LbrM.10.1580  LbrM.10.1690 |  |  |  |
|  |  |  |  |  |  |
| **serine protease** | katanin-like protein | LbrM.13.0770; LbrM.28.0410 |  |  |  |
|  |  |  |  |  |  |
|  | rhomboid-like protein | LbrM.02.0310 |  |  |  |
|  | subtilisin-like serine peptidase | LbrM.13.0860  (*) The sequences were clustered using the software CD-HIT and the cutoff used was 80% of identity. |  |  |  |

(*) The sequences were clusterized using the software CD-HIT and the cutoff used was 80% of identity.

(*) The sequences were clusterized using the software CD-HIT and the cutoff used was 80% of identity.

| **Supplementary table 2: Cluster of genes Leishmania spp** | | |  | Cysteine protease | | |  | Serine protease | | |
| --- | --- | --- | --- | --- | --- | --- | --- | --- | --- | --- |
| **Base pair*** | **Gene**** | **ID (%)***** |  | **Base pair*** | **Gene**** | **ID (%)***** |  | **Base pair*** | **Gene**** | **ID (%)***** |
| 4455 ± 2.4 | LbrM.33.0210 LinJ.33.0210 LmjF.33.0200 LmxM.32.0200 | 82 ± 0..3 |  | 4667 ± 8 | LbrM.20.0290 LinJ.34.0300 LmjF.34.0280 LmxM.33.0280 | 84 ± 0.3 |  | 5210 ± 4.5 | LbrM.28.2570 LinJ.28.2540 LmjF.28.2380 LmxM.28.2380 LmxM.32.0400 | 93 ± 6.4 |
| 4109 ± 41.7 | LbrM.12.0930 LinJ.12.0830 LmjF.12.1250 LmxM.12.1250 | 87 ± 6.0 |  | 4040 ± 31 | LbrM.32.3160 LinJ.32.3060 LmjF.32.2910 LmxM.31.2910 | 86 ± 0.7 |  | 4204 ± 899 | LbrM.27.0470 LinJ.27.0390 LmjF.27.0380 LmxM.27.0380 | 85 ± 0.9 |
| 3099 ± 0 | LbrM.07.0100 LinJ.07.0250 LmjF.07.0100 LmxM.07.0100 | 89 ± 0.1 |  | 3495 ± 0 | LbrM.34.1640 LinJ.35.1730 LmjF.35.1740 LmxM.34.1740 | 88 ± 0.3 |  | 2718 ± 0 | LbrM.06.0320 LinJ.06.0340 LmjF.06.0340 LmxM.06.0340 | 91 ± 0.2 |
| 2767 ± 218 | LbrM.31.0870 LinJ.31.0730 LmjF.31.0700 LmxM.30.0700 | 85 ± 0.2 |  | 3392 ± 7 | LbrM.30.0260 LinJ.30.0250 LmjF.30.0250 LmxM.29.0250 | 95 ± 3.5 |  | 2558 ± 1.5 | LbrM.35.2630 LinJ.36.2550 LmjF.36.2420 LmxM.36.2420 | 92 ± 4.3 |
| 2653 ± 14 | LbrM.26.0330 LinJ.26.0290 LmjF.26.0300 LmxM.26.0300 | 90 ± 5.4 |  | 3021 ± 0 | LbrM.30.1320 LinJ.30.1260 LmjF.30.1200 LmxM.29.1200 | 82 ± 0.4 |  | 2192 ± 8.5 | LbrM.09.0850 LinJ.09.0820 LmjF.09.0770 LmxM.09.0770 | 87 ± 0.7 |
| 2607 ± 0 | LbrM.29.2220 LinJ.29.2350 LmjF.29.2240 LmxM.08.29.2240 | 84 ± 0.4 |  | 2712 ± 0 | LbrM.20.3690 LinJ.34.3890 LmjF.34.4060 LmxM.33.4060 | 92 ± 4.7 |  | 1867 ± 9.1 | LbrM.20.6000 LinJ.12.0920 LmjF.12.1330 LmxM.12.1330 | 89 ± 6.0 |
| 2000 ± 117 | LbrM.26.1590 LinJ.26.1550 LmjF.26.1570 LmxM.26.1570 | 93 ± 4.4 |  | 2468 ± 130 | LbrM.31.0510 LinJ.31.0410 LmjF.31.0390 LmxM.30.0390 | 89 ± 1.8 |  | 1084 ± 160 | LbrM.04.0810 LinJ.04.0850 LmjF.04.0850 LmxM.04.0850 | 93 ± 3.0 |
| 1998 ± 251 | LbrM.20.1030 LinJ.34.1130 LmjF.34.1060 LmxM.33.1060 | 91 ± 0.3 |  | 2201 ± 94 | LbrM.31.0620 LinJ.31.0480 LmjF.31.0460 LmxM.30.0460 | 91 ± 5.3 |  | 1191 ± 0 | LbrM.03.0450 LinJ.03.0520 LmjF.03.0540 LmxM.03.0540 | 91 ± 0.2 |
| 2040 ± 0 | LbrM.05.0940 LinJ.05.0960 LmjF.05.0960 LmxM.05.0960 | 90 ± 0.2 |  | 2303 ± 19 | LbrM.18.1160 LinJ.18.1070 LmjF.18.1060 LmxM.18.1060 | 93 ± 1.9 |  | 1185 ± 0 | LbrM.34.4000 LinJ.35.4070 LmjF.35.4020 LmxM.34.4020 | 86 ± 0.2 |
| 2031 ± 0 | LbrM.35.4690 LinJ.36.4670 LmjF.36.4450 LmxM.36.4450 | 88 ± 0.1 |  | 2249 ± 4.5 | LbrM.29.2270 LinJ.29.2410 LmjF.29.2300 LmxM.08.29.2300 | 86 ± 0.5 |  | 1012 ± 7.5 | LbrM.09.0680 LinJ.09.0650 LmjF.09.0600 LmxM.09.0600 | 85 ± 0.7 |
| 1797 ± 0 | LbrM.18.0690 LinJ.18.0620 LmjF.18.0610 LmxM.18.0610 | 89 ± 0.4 |  | 2232 ± 0 | LbrM.20.5410 LinJ.20.1240 LmjF.20.1200 LmxM.20.1200 | 87 ± 0.5 |  | 674 ± 7.5 | LbrM.35.0280 LinJ.36.0220 LmjF.36.0200 LmxM.36.0200 | 90 ± 6.5 |
| 1797 ± 0 | LbrM.19.1850 LinJ.19.1620 LmjF.19.1590 LmxM.19.1590 | 90 ± 0.1 |  | 2119 ± 1.7 | LbrM.25.1350 LinJ.25.1540 LmjF.25.1480 LmxM.25.1480 | 93 ± 5.1 |  |  |  |  |
| 1688 ± 57 | LbrM.35.2900 LinJ.36.2850 LmjF.36.2710 LmxM.36.2710 | 96 ± 3.0 |  | 2064 ± 0 | LbrM.20.5400 LinJ.20.1230 LmjF.20.1190 LmxM.20.1190 | 90 ± 0.3 |  |  |  |  |
| 1615 ± 165 | LbrM.23.1030 LinJ.23.1120 LmjF.23.0950 LmxM.23.0950 | 93 ± 4.1 |  | 1728 ± 1.5 | LbrM.27.1390 LinJ.27.1170 LmjF.27.1270 LmxM.27.1270 | 94 ± 7.3 |  |  |  |  |
| 1586 ± 1.5 | LbrM.13.0680 LinJ.13.0760 LmjF.13.0870 LmxM.13.0870 | 92 ± 5.4 |  | 1406 ± 72 | LbrM.31.0140 LinJ.31.0150 LmjF.31.0140 LmxM.30.0140 | 87± 0.2 |  |  |  |  |
| 1584 ± 0 | LbrM.33.2840 LinJ.33.2700 LmjF.33.2570 LmxM.32.2570 | 92 ± 5.3 |  | 1326 ± 18 | LbrM.34.1490 LinJ.35.1580 LmjF.35.1580 LmxM.34.1580 | 87 ± 4.0 |  |  |  |  |
| 1512 ± 0 | LbrM.13.0080 LinJ.13.0090 LmjF.13.0090 LmxM.13.0090 | 85 ± 0.3 |  | 1332 ± 76 | LinJ.08.0950 LinJ.08.0960 LmjF.08.1030 LmjF.08.1060 LmjF.08.1080 LmxM.08.1070 | 87 ± 0.5 |  |  |  |  |
| 1499 ± 1.5 | LbrM.33.2810 LinJ.33.2670 LmjF.33.2540 LmxM.32.2540 | 92 ± 5.2 |  | 1185 ± 6 | LbrM.30.0280 LinJ.30.0270 LmjF.30.0270 LmxM.29.0270 | 87 ± 0.1 |  |  |  |  |
| 1473 ± 0 | LbrM.34.1300 LinJ.35.1390 LmjF.35.1380 LmxM.34.1380 | 92 ± 0.1 |  | 1167 ± 15 | LbrM.32.4130 LinJ.32.4040 LmjF.32.3890 LmxM.31.3890 | 82 ± 1.0 |  |  |  |  |
| 1426 ± 7.9 | LbrM.16.0860 LinJ.16.0850 LmjF.16.0850 LmxM.16.0850 | 91 ± 6.5 |  | 1058 ± 25 | LbrM.18.0380 LinJ.18.0360 LmjF.18.0360 LmxM.18.0360 | 82 ± 0.4 |  |  |  |  |
| 1404 ± 0 | LbrM.21.0400 LinJ.21.0400 LmjF.21.0340 LmxM.21.0340 | 89 ± 0.3 |  | 924 ± 0 | LbrM.24.0420 LinJ.24.0420 LmjF.24.0420 LmxM.24.0420 | 89 ± 1.0 |  |  |  |  |
| 1362 ± 0 | LbrM.29.2330 LinJ.29.2470 LmjF.29.2360 LmxM.08.29.2360 | 84 ± 0.6 |  | 811 ± 4.5 | LbrM.17.1540 LinJ.17.1520 LmjF.17.1400 LmxM.17.1400 | 92 ± 6.4 |  |  |  |  |
| 1284 ± 0 | LbrM.27.0050 LinJ.27.0040 LmjF.27.0040 LmxM.27.0040 | 81 ± 0.2 |  | 702 ± 0 | LbrM.25.0190 LinJ.25.0190 LmjF.25.0190 LmxM.25.0190 | 82 ± 0.2 |  |  |  |  |
| 1272 ± 0 | LbrM.17.0180 LinJ.17.0250 LmjF.17.0140 LmxM.1.0140 | 85 ± 0.8 |  | 457 ± 1.7 | LbrM.20.5500 LinJ.20.1320 LmjF.20.1280 LmxM.20.1280 | 93 ± 5.7 |  |  |  |  |
| 1170 ± 72 | LbrM.29.1660 LinJ.29.1680 LmjF.29.1570 LmxM.08.29.1570 | 88 ± 0.3 |  | 441 ± 0 | LbrM.20.5440 LinJ.20.1260 LmjF.20.1220 LmxM.20.1220 | 84.8± 0.4 |  |  |  |  |
| 1143 ± 0 | LbrM.19.0470 LinJ.19.0150 LmjF.19.0160 LmxM.19.0160 | 89 ± 0.1 |  | 348 ± 0 | LbrM.14.0820 LbrM.14.0830 LinJ.04.0430 LinJ.14.0910 LinJ.14.0920 LmjF.14.0850 LmxM.14.0851 | 92 ± 3.8 |  |  |  |  |
| 1095 ± 0 | LbrM.31.0100 LinJ.31.0110 LmjF.31.0100 LmxM.30.0100 | 89 ± 0.6 |  |  |  |  |  |  |  |  |
| 920 ± 109 | LbrM.26.2360 LinJ.26.2450 LmjF.26.2430 LmxM.26.2430 | 89 ± 6.5 |  |  |  |  |  |  |  |  |

*: mean and standard deviation (±) of the numbers of base pairs in cluster

**: protease genes in cluster

***: identity to consensus sequences (ID) in percentage
